# Predicting phenotypes of asthma and eczema with machine learning

**DOI:** 10.1186/1755-8794-7-S1-S7

**Published:** 2014-05-08

**Authors:** Mattia CF Prosperi, Susana Marinho, Angela Simpson, Adnan Custovic, Iain E Buchan

**Affiliations:** 1 Centre for Health Informatics, Institute of Population Health, Faculty of Medical and Human Sciences, University of Manchester, Manchester, UK; 2 Centre for Respiratory Medicine and Allergy, Institute of Inflammation and Repair, University of Manchester, Manchester, UK

**Keywords:** Asthma, wheeze, eczema, allergen, lung function, single nucleotide polymorphisms, machine learning, model selection, diagnostics

## Abstract

**Background:**

There is increasing recognition that asthma and eczema are heterogeneous diseases. We investigated the predictive ability of a spectrum of machine learning methods to disambiguate clinical sub-groups of asthma, wheeze and eczema, using a large heterogeneous set of attributes in an unselected population. The aim was to identify to what extent such heterogeneous information can be combined to reveal specific clinical manifestations.

**Methods:**

The study population comprised a cross-sectional sample of adults, and included representatives of the general population enriched by subjects with asthma. Linear and non-linear machine learning methods, from logistic regression to random forests, were fit on a large attribute set including demographic, clinical and laboratory features, genetic profiles and environmental exposures. Outcome of interest were asthma, wheeze and eczema encoded by different operational definitions. Model validation was performed via bootstrapping.

**Results:**

The study population included 554 adults, 42% male, 38% previous or current smokers. Proportion of asthma, wheeze, and eczema diagnoses was 16.7%, 12.3%, and 21.7%, respectively. Models were fit on 223 non-genetic variables plus 215 single nucleotide polymorphisms. In general, non-linear models achieved higher sensitivity and specificity than other methods, especially for asthma and wheeze, less for eczema, with areas under receiver operating characteristic curve of 84%, 76% and 64%, respectively. Our findings confirm that allergen sensitisation and lung function characterise asthma better in combination than separately. The predictive ability of genetic markers alone is limited. For eczema, new predictors such as bio-impedance were discovered.

**Conclusions:**

More usefully-complex modelling is the key to a better understanding of disease mechanisms and personalised healthcare: further advances are likely with the incorporation of more factors/attributes and longitudinal measures.

## Background

Asthma is the most common chronic disease in developed countries, however, the drug armamentarium available to manage the condition is modest[1]. There is increasing recognition that asthma is a heterogeneous disease with multiple endotypes, which may have similar clinical manifestations, or phenotypes, but different underlying pathophysiological causes[2,3]. Appropriate identification of such endotypes is critical for the understanding of the disease mechanism and the development of personalised approaches to its management[4]. Sensitisation to allergens from several sources (such as pets, dust mites, cockroaches, and pollens) has been independently associated with asthma and asthma-related symptoms[5-8], and among asthmatic patients with the severity of the disease[9-12]. Also, sensitisation to inhalant allergens has been found to be associated with diminished lung function and increased airway responsiveness[13].

It remains unclear to what extent allergen sensitisation and lung function markers (e.g., airway reactivity, airway inflammation), in conjunction with a broader set of other potentially relevant information (e.g. environmental exposures or genetic characteristics), contribute towards specific clinical manifestations of different atopic diseases (e.g. asthma *vs*. eczema). In the past decades, several approaches to predict such current or subsequent clinical manifestations, both in children and adults, have been introduced[14-22]. The performance of prediction models varies in relation to different population strata, and obviously in relation to the clinical outcome or end-point definitions. For instance, one of the earliest works, by Castro-Rodríguez *et al*.[14], devised a rule-based asthma predictive index to predict subsequent asthma amongst young children with a history of wheezing, attaining sensitivity ~0.4 at ~0.8 specificity on various time points. The recent work by Chatzimichail *et al*.[23] reported ~0.95 of both sensitivity and specificity in predicting current asthma in symptomatic preschool children, using a machine learning approach based on previous symptoms, medications, allergen sensitisation and lung function.

In this work, using a rich data set from an unselected cross-sectional population study, different operational definitions of current asthma, wheeze and eczema are carefully derived, and we analyse their prognostic factors from a large set of markers, which includes demographic, clinical, laboratory features, genetic profiles and environmental exposures. Of note, previous diagnoses (along with anti-asthma medication usage) are removed on purpose from the input set, as many clinical outcome definitions are recursively based on them. The aim is to identify to which extent such heterogeneous information contributes and combines towards the prediction of a specific clinical presentation - comparing linear and non-linear machine learning models fitted with different feature combinations - and eventually prepare the grounds for the deployment of a personalized diagnostic tool.

## Methods

The study population comprised a cross-sectional sample of adult individuals, age ≥18 years, including representatives of the general population enriched by subjects with asthma[13,24]. For the sample from the general population, we approached parents of children who have been under active follow-up in the Manchester Asthma and Allergy Study (population-based birth cohort study)[25]. The population of subjects with asthma included well-phenotyped adults who were identified from a clinical trials database, and had both a history of physician-diagnosed asthma and asthma symptoms within the previous 12 months[26,27]. The study was approved by the Local Research Ethics Committee (05/Q1406/70) and is registered as N0226171141. Written informed consent was obtained from all subjects.

A total of 1,102 attributes of the study participants were collected across a large heterogeneous information spectrum, including interviewer-administered questionnaires, laboratory measurements, doctors' diagnoses and environmental exposures. The collected data included:

• demographic information (e.g. gender, ethnicity, age, place of residence);

• questionnaire data related to symptom presence and severity (e.g. wheeze, shortness of breath, chronic cough), previous/current diagnoses of asthma, hay fever, eczema, food allergies or other illnesses;

• use of anti-asthma medications (e.g. short-acting beta agonists [SABA], long-acting beta agonists [LABA], inhaled corticosteroids [ICS]);

• questionnaire data on smoking and alcohol drinking habits, current pet ownership, indoor environmental conditions (e.g. rugs, beds, type of house heating, latex usage), occupation and occupation-related accidents;

• objective measures on environmental exposure to house dust mite (Der p 1), cat (Fel d 1) and dog (Can f 1) allergen determined in dust samples collected from homes using enzyme-linked immunosorbent assays (ELISAs);

• objective measures on environmental exposure to endotoxin (marker of exposure to gram-negative bacteria) and beta glucan (marker of exposure to moulds) determined in dust samples collected from homes;

• body measurements (e.g. height, weight, body mass index [BMI], fat percentage, whole body impedance);

• lung function measurements (e.g. forced expiratory volume in 1 second [FEV_1_], forced vital capacity [FVC], peak expiratory flow [PEF], functional reserve capacity [FRC] and residual volume [RV], total lung capacity [TLC], forced expiratory flow 25-75% [FEF_25-75_] and specific airway resistance [sRaw]);

• measurement of airway inflammation (exhaled nitric oxide [eNO])

• measurement of airway hyper-responsiveness using methacholine challenge, expressed as a provocative concentration of methacholine needed to produce a 20% fall in FEV_1 _(PC20), and methacholine dose-response slope (MDRS);

• assessment of atopic status using (i) skin prick tests (SPT), (ii) measurement of serum allergen-specific Immunoglobulin E values (IgE), and (iii) component resolved diagnostics using an immuno-dot blot as previously described[28].

In addition, as part of a candidate gene association study, subjects were genotyped for 215 single nucleotide polymorphisms (SNPs) in genes found to be associated with asthma in previous studies (including polymorphisms in chromosomal regions 20p13-p12 and 17q12-21)[24].

We used the following (partly overlapping) definitions of asthma, wheeze and eczema.

*Asthma *was encoded with three "operational definitions" determined by questionnaire, specifically:

i. current asthma (CA), based on De Marco *et al*.[29], defined as asthma ever confirmed by a doctor and at least one symptom of wheeze, nocturnal chest tightness, asthma attack within the past 12 months, attacks of breathlessness following activity, at rest or at night-time, having taken anti-asthma medication;

ii. level-2 ECRHS II[30] definition (A2), as two positive answers to the questions "have you been woken by an attack of shortness of breath at any time in the last 12 months", "have you had an attack of asthma in the last 12 months", "are you currently taking any medicines including inhalers, aerosol or tablets for asthma";

iii. level-3 ECRHS II definition (A3), as three positive answers out of the set described at the previous point.

*Current wheeze *(CW) was defined, according to Pekkanen *et al*.[31], as the presence of wheeze/breathlessness in the previous 12 months outside colds.

*Eczema *was defined as self-diagnosed (SDE) or doctor-confirmed (DDE) eczema.

Out of the 1,102 original non-genetic attributes, 223 were selected by clinical researchers, excluding factors considered as irrelevant or completely redundant, and those that were defining features of diagnoses. Attributes were grouped into: demographic/environmental variables (n = 74, including age, gender, BMI, whole body impedance, housing conditions, pet ownership, plus n = 56 variables measuring environmental exposures to endotoxin, beta glucan and indoor allergens); lung function, airway inflammation and airway hyper-responsiveness markers (n = 12, including eNO, % predicted FEV_1_, FVC, FEV_1_/FVC, FEF_25-75_, sRaw, PEF, TLC, RV, methacholine challenge MDRS and PC20); allergen sensitization assessed either by skin prick testing, specific serum IgE measurement or component resolved diagnostics (n = 8, n = 7, n = 66, respectively), recording mean wheal diameters (MWD) and IgE levels, which were either log-transformed or discretized into ordered quartile categories (where a negative or below limit of detection result was the zero-order category). All 215 SNPs were retained and merged to the data set. Before data merge, raw SNP data were processed through linkage-disequilibrium filtering/imputation using Haploview[32] and the method of Gabriel *et al*.[33] (as described in the previous work by Marinho *et al*.[24]). Other missing values were replaced by column-wise median and modes depending on the data types.

For descriptive statistics and comparison with other prediction methods, information about previous diagnoses and medication usage (ICS, SABA, LABA) was retained but not used as input for the main models.

Main-effects logistic regression (LR) models were fitted selecting features by means of the LogitBoost algorithm[34]. For comparison purposes, a LR model made by the best single predictor according to the Akaike information criterion (named one rule, OR) was considered[35]. A decision tree model (DT)[36] and a decision tree ensemble, the random forest (RF, 250 trees)[37] were also evaluated, along with the AdaBoost (AB) classifier[38]. Goodness-of-fit functions examined were: accuracy, i.e. percentage of correctly classified cases; area under the receiver operating characteristic curve (AUROC), which is equal to the probability that a classifier ranks a randomly chosen positive instance (e.g. condition present/diagnosed) higher than a randomly chosen negative one (e.g. condition absent); sensitivity, i.e. the probability that the classification is positive when the condition is present (true positive rate); specificity, i.e. the probability that the classification is negative when the condition is not present (true negative rate). Model performance was estimated and compared as extra-sample via bootstrapping (100 replicates), considering out-of-bag distributions, and assessing significance via t-tests adjusted for sample overlap and multiple comparisons[39-41]. Attribute importance was assessed by means of RF, calculating the average re-scaled (i.e. divided by its standard error) decrease in accuracy by variable randomization (repeated for 1000 times), and comparing it against a null distribution obtained by shuffling outcome labels, calculating p-values according to the method of Altmann *et al*.[42] and previous works[43,44]. All analyses were carried out using R software (http://www.r-project.org/).

## Results

### Characteristics of the study population

The study population included 554 subjects, with a mean (standard deviation) age of 43 (5) years at the time of the assessment, 42% male, 38% previous or current smokers. The proportion of CA, CW and DDE were 16.7%, 12.3%, and 21.7%, respectively. Subjects' characteristics are described in detail in Table 1, as well as cross-tabulation of outcomes. There was a high level of agreement between SDE and DDE (95.5%), as well as between CA, A2, A3 and CW (from 95.3% of CA vs. A2 to 87.7% of CA vs. A3), as expected by their intersecting definitions. The lowest agreement was found between SDE and CW (73.4%). For ease of reading, we have omitted information about genetic data, which has been described in detail previously[24] (available upon request). Of note, 73.2% attributes had no missing data, and the amount of missingness in the rest was a median (interquartile range, IQR) of 0.7% (0.2%-2.2%).

**Table 1:** Study data

variable				median (IQR)		#missing (%)
age (years)				42.6 (39.7-45.7)		0 (0%)

year of birth				1964 (1961-1967)		0 (0%)

body mass index (BMI)				26 (23.6-29.1)		2 (0.4%)

whole body impedance				613.5 (550-685)		34 (6.1%)

fat %				29.5 (23.7-36)		34 (6.1%)

exhaled nitric oxide (eNO), ppb (log_e _scale)				2.8 (2.4-3.3)		94 (17.0%)

specific airway resistance (sRaw), kPa/s (log_e _scale)				-0.1 (-0.3-0.1)		11 (2.0%)

peak expiratory flow (PEF) % predicted				113.1 (102.1-124.6)		9 (1.6%)

forced vital capacity (FVC) % predicted				114.5 (105.6-123.1)		11 (2.0%)

forced expiratory volume in 1 second (FEV^ % predicted				106 (98.6-115.5)		10 (1.8%)

forced expiratory flow (FEF_25-75_) % predicted				80 (66-96.3)		11 (2.0%)

total lung capacity (TLC)				108.5 (101-116.9)		11 (2.0%)

residual volume (RV)				113 (98.6-127.8)		11 (2.0%)

FEVj/FVC ratio				0.8 (0.8-0.8)		11 (2.0%)

provocative concentration of methacholine needed to produce a 20% fall in FEVj (PC20), of those completing the test				5.3 (1.2-9.0)		43 (7.8%)

methacholine dose-response slope (MDRS), transformed as 100/(MdRS+10)				5.7 (4.2-7.5)		43 (7.8%)

**variable**				**N (%)**		**#missing (%)**

Gender		male		234 (42.2%)		0 (0%)

smoking status		never		341 (61.6%)		0 (0%)
		
		ex-smoker		144 (26%)		0 (0%)
		
		current		69 (12.5%)		0 (0%)

cat/dog ownership				186 (33.6%)		1 (0.2%)

allergen sensitisation by skin prick test (SPT)		dust mite (mean wheal diameter >3 mm)		162 (29.3%)		1 (0.2%)
		
		cat (mean wheal diameter >3 mm)		106 (19.2%)		1 (0.2%)
		
		dog (mean wheal diameter >3 mm)		48 (8.6%)		1 (0.2%)
		
		tree (mean wheal diameter >3 mm)		76 (13.8%)		1 (0.2%)
		
		grass (mean wheal diameter >3 mm)		129 (23.4%)		1 (0.2%)
		
		mould (mean wheal diameter >3 mm)		16 (2.9%)		1 (0.2%)
		
		peanut (mean wheal diameter >3 mm)		9 (1.7%)		1 (0.2%)

bird ownership				13 (2.4%)		1 (0.2%)

medications in the past three months		short-acting beta agonists (SABA)		34 (6.1%)		1 (0.2%)
		
		inhaled corticosteroids (ICS) or ICS/long-acting beta agonists (LABA)		37 (6.7%)		1 (0.2%)

illness or problem caused by eating a particular food or foods, ever				97 (17.5%)		1 (0.2%)

accident at home, work or elsewhere exposing to high levels of vapours, gas or dust				22 (4%)		2 (0.4%)

carpets in the house				292 (52.8%)		1 (0.2%)

gas stove in the house				432 (78.1%)		1 (0.2%)

electric stove in the house				231 (41.8%)		1 (0.2%)

job causing wheezing problems				33 (6%)		3 (0.5%)

proportion of subjects not completing PC20				423 (82.8%)		43 (7.8%)

proportion of subjects with current asthma (CA)				93 (16.7%)		0 (0%)

proportion of subjects with level-2 asthma (A2)				70 (12.7%)		0 (0%)

proportion of subjects with level-3 asthma (A3)				24 (4.3%)		0 (0%)

proportion of subjects with current wheeze (CW)				68 (12.3%)		0 (0%)

proportion of subjects with self-diagnosed eczema (SDE)				146 (26.3%)		0 (0%)

proportion of subjects with doctor's diagnosed eczema (DDE)				120 (21.7%)		0 (0%)

**cross-tabulation of clinical outcomes (% of agreement)**						

	SDE	DDE	CA	A2	CW	A3

SDE		95.47%	74.82%	74.82%	73.37%	74.09%

DDE	95.47%		77.17%	77.90%	76.09%	78.26%

CA	74.82%	77.17%		95.29%	89.49%	87.68%

A2	74.82%	77.90%	95.29%		92.39%	91.67%

CW	73.37%	76.09%	89.49%	92.39%		90.94%

A3	74.09%	78.26%	87.68%	91.67%	90.94%	

Characteristics of the study population (n = 554) and cross-tabulation of outcomes.

## Model inference

**Figure 1:**
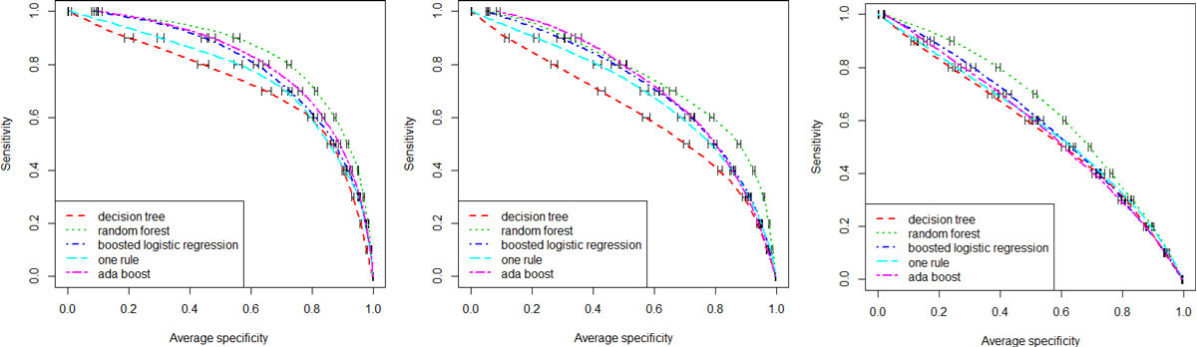
**Comparison of machine learning methods**. Performance comparison of different machine learning techniques in terms of area under the receiver operating characteristic curve in predicting current asthma (left panel), current wheeze (middle panel), and doctor's diagnosed eczema (right panel) using the whole feature set (demographic, environmental, genetic, lung function markers, and allergen sensitization). Results are out-of-bag predictions averaged over 100 bootstrap runs.

Given the levels of agreement between outcomes, inference results will be presented here for CA, CW and DDE (results were similar for the other outcomes and are available upon request). Different learning methods (LR, RF, AB, DT, OR) were applied to the full attribute set with the intent to identify both the best performing model (in terms of AUROC, sensitivity and specificity, given the high class imbalance of all outcomes) and to evaluate if non-linear models were able to improve the goodness-of-fit as compared to main-effects LR. RF consistently yielded the most predictive models (average AUROCs for CA, CW and DDE were 84%, 76%, and 64%, respectively), but the difference in mean AUROC with respect to LR and AB across 100 bootstrap runs could not be considered significantly shifted from zero at the 0.05 level. Instead, OR and DT were consistently superseded by RF. Figure 1 plots averaged ROC curves for the three different outcomes of CA, DDE and CW, whilst Table 2 shows averaged AUROC, and specificity at different sensitivity levels, both estimated from out-of-bag distributions across 100 bootstrap runs.

**Table 2:** Comparison of machine learning methods.

outcome	Model	AUROC	sensitivity (at 90% specificity)	sensitivity (at 80% specificity)	accuracy
Doctor's Diagnosed Eczema	Decision Tree*	0.57 (0.04)	0.15 (0.07)	0.29 (0.07)	0.78 (0.02)
	
	Random Forest	0.64 (0.03)	0.2 (0.06)	0.34 (0.07)	0.79 (0.02)
	
	Logistic Regression	0.59 (0.04)	0.18 (0.06)	0.31 (0.08)	0.78 (0.02)
	
	One Rule*	0.58 (0.06)	0.2 (0.11)	0.3 (0.15)	0.79 (0.02)
	
	AdaBoost	0.58 (0.04)	0.17 (0.06)	0.3 (0.07)	0.78 (0.02)

Current Asthma	Decision Tree*	0.72 (0.06)	0.39 (0.12)	0.54 (0.11)	0.85 (0.02)
	
	Random Forest	0.84 (0.03)	0.55 (0.09)	0.72 (0.08)	0.87 (0.02)
	
	Logistic Regression	0.79 (0.04)	0.45 (0.08)	0.63 (0.08)	0.86 (0.02)
	
	One Rule*	0.76 (0.06)	0.44 (0.09)	0.61 (0.11)	0.86 (0.02)
	
	AdaBoost	0.81 (0.04)	0.48 (0.09)	0.66 (0.07)	0.86 (0.02)

Current Wheeze	Decision Tree*	0.62 (0.06)	0.27 (0.1)	0.36 (0.11)	0.88 (0.02)
	
	Random Forest	0.76 (0.04)	0.47 (0.09)	0.6 (0.09)	0.89 (0.02)
	
	Logistic Regression	0.72 (0.04)	0.34 (0.08)	0.51 (0.08)	0.88 (0.02)
	
	One Rule*	0.69 (0.06)	0.33 (0.09)	0.49 (0.12)	0.88 (0.02)
	
	AdaBoost	0.73 (0.04)	0.32 (0.09)	0.5 (0.09)	0.88 (0.02)

Performance of machine learning models on different outcomes using the full set of demographic, environmental, genetic (single nucleotide polymorphisms), allergen sensitisation, and lung functions variables. Results are mean (standard deviation) values estimated from out-of-bag distributions across 100 bootstrap runs.

^*^difference in AUROC significantly shifted from zero at the 0.05 level as compared to that of a random forest. AUROC: area under the receiver operating characteristic curve.

**Figure 2:**
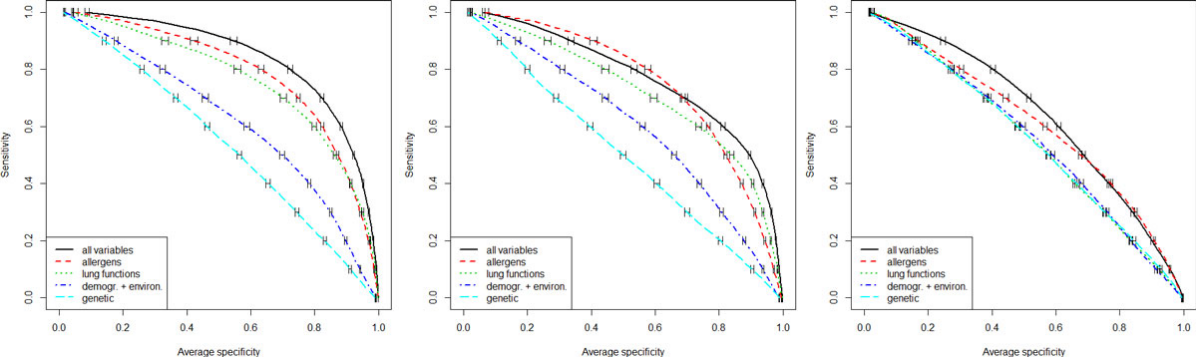
**Comparison of random forest performance using selected input domains**. Performance comparison of random forests in terms of area under the receiver operating characteristic curve in predicting current asthma (left panel), current wheeze (middle panel), and doctor's diagnosed eczema (right panel) using the whole feature set (demographic, environmental, genetic, lung function markers, and allergen sensitization) and selected feature subsets. Results are out-of-bag predictions averaged over 100 bootstrap runs.

Based on these results, the RF method was retained and tested using different subsets of the original variable space - specifically the groups of allergen sensitisation, lung functions/airway hyper-responsiveness, demographic/environmental variables, and genetic variants (as defined in the methods) - in order to identify to which extent each group was contributing to increasing AUROC, sensitivity or specificity with respect to each outcome prediction. Table 3 shows averaged AUROC, and specificity at different sensitivity levels, both estimated from out-of-bag distributions across 100 bootstrap runs. For all outcomes, RF models using the allergen sensitisation yielded the best performance, followed in order by lung functions, demographic/environment, and genetic SNPs variable subsets. Usage of the whole variable set increased AUROC over each of the subsets for all outcomes (below the 0.05 significance level for demographic/environment and genetic feature subsets when predicting CA and CW). Figure 2 shows receiver operating characteristic curves for the three outcomes considering each different feature subset, averaged across 100 bootstrap runs.

**Table 3:** Comparison of random forest performance using selected input domains.

outcome	feature set	AUROC	p-value*	sensitivity (at 90% specificity)	sensitivity (at 80% specificity)	accuracy
Doctor's Diagnosed Eczema	allergens	0.62 (0.03)	0.34	0.22 (0.06)	0.37 (0.06)	0.79 (0.02)
	
	lung functions	0.56 (0.04)	0.08	0.13 (0.05)	0.24 (0.06)	0.78 (0.02)
	
	genetic	0.56 (0.04)	0.11	0.14 (0.05)	0.25 (0.06)	0.78 (0.02)
	
	demographic/environ.	0.56 (0.04)	0.05	0.12 (0.05)	0.24 (0.07)	0.78 (0.02)
	
	all	0.65 (0.04)	reference	0.2 (0.07)	0.35 (0.08)	0.79 (0.02)

Current Asthma	allergens	0.79 (0.04)	0.11	0.43 (0.08)	0.64 (0.07)	0.86 (0.02)
	
	lung functions	0.76 (0.04)	0.04	0.44 (0.08)	0.6 (0.09)	0.86 (0.02)
	
	genetic	0.54 (0.04)	<0.0001	0.12 (0.05)	0.23 (0.07)	0.83 (0.02)
	
	demographic/environ.	0.62 (0.04)	<0.0001	0.2 (0.08)	0.38 (0.07)	0.83 (0.02)
	
	all	0.84 (0.03)	reference	0.56 (0.09)	0.73 (0.08)	0.87 (0.02)

Current Wheeze	allergens	0.75 (0.04)	0.35	0.34 (0.09)	0.54 (0.1)	0.88 (0.02)
	
	lung functions	0.72 (0.05)	0.19	0.42 (0.09)	0.55 (0.08)	0.89 (0.02)
	
	genetic	0.5 (0.05)	0.0002	0.11 (0.06)	0.21 (0.08)	0.88 (0.02)
	
	demographic/environ.	0.6 (0.05)	0.006	0.17 (0.07)	0.32 (0.09)	0.88 (0.02)
	
	all	0.77 (0.04)	reference	0.5 (0.09)	0.62 (0.07)	0.89 (0.02)

Performance of random forest on different outcomes using specific variable subsets and the full set of demographic, environmental, genetic (single nucleotide polymorphisms), allergen sensitisation, and lung functions variables. Results are mean (standard deviation) estimated from out-of-bag distributions across 100 bootstrap runs.

^*^testing the hypothesis of difference in AUROC significantly shifted from zero as compared to that of a random forest model using all variables with a corrected paired t-test.

AUROC: area under the receiver operating characteristic curve.

**Figure 3:**
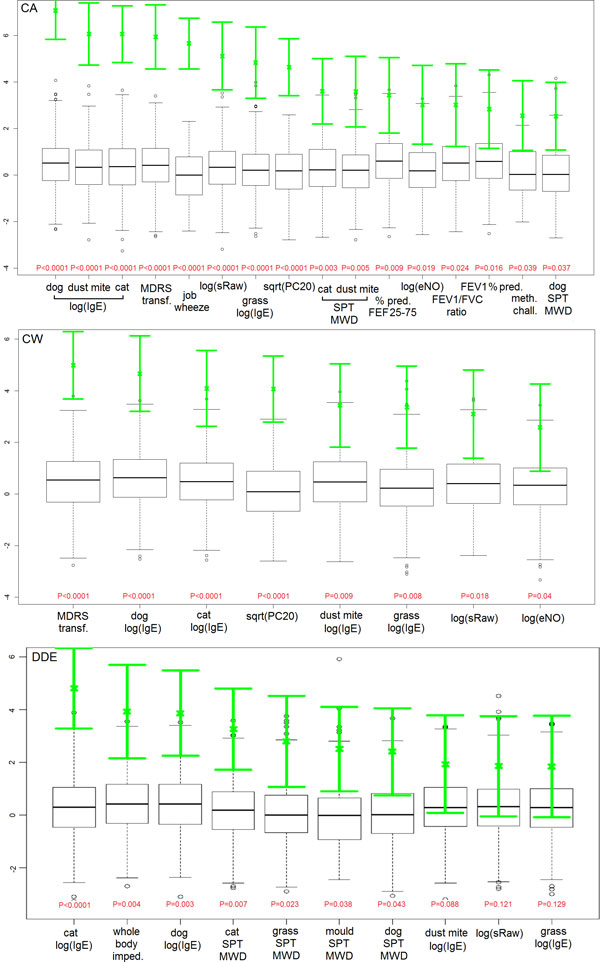
**Feature importance evaluation by means of random forests**. Importance is calculated and shown as the rescaled mean (standard deviation) decrease in accuracy over 1000 independent runs (green colour). Boxplots represent a null feature importance distribution obtained by permuting the outcome randomly for 1000 times. Variables significant at the 0.1 level (p-values in red) are shown for current asthma (upper panel), current wheeze (middle panel), and doctor's diagnosed eczema (lower panel) using the whole feature set as input.

When looking at feature importance, the most relevant attributes reflected the overall performance of RF trained using feature subsets: variables from allergen sensitisation and lung function groups were the highest ranked, especially for CA and CW, as shown in Figure 3. Of note, when considering DDE, whole body impedance was the second most important variable reported by RF, and this was confirmed by LR, showing to a higher risk of DDE (OR 1.19 per square root increase, 95% CI 1.13-1.27, p-value = 0.0016), and found to be correlated to BMI (Pearson's ρ=-0.54, p-value<0.0001). No SNPs were scored within the 25^th ^percentile of RF importance, but crude associations with asthma symptoms were confirmed. When considering CW, rs4986790 was the SNP with highest level of association using a chi-square test on allele categories (unadjusted p = 0.005), whilst rs6037651 using the additive model (unadjusted p = 0.003). When considering DDE, rs2569190 (unadjusted p = 0.003 from chi-square) and rs574174 (unadjusted p = 0.004 from additive model). However their significance was not below the 0.05 level when correcting for multiple testing (using Benjamini-Hochberg correction). For CA, rs7212938 and rs8079416 were the top-scoring SNPs under the categorical and additive model, respectively (unadjusted p = 0.0002 and p = 0.0006), and the significance remained below the 0.1 level after adjusting for multiple testing (p = 0.09 and p = 0.03).

To compare more thoroughly RF with LR, we analysed the variable sets selected by the LogitBoost algorithm. Specifically, for CW, five predictors were selected: IgE of house dust mite (OR = 1.207 per log_e _higher, p = 0.005); IgE of dog (OR = 1.465 per log_e _higher, p < 0.0001); number of cigarettes smoked (OR = 1.032 per packages/year, p = 0.003); moving house (OR = 3.078 for moving twice or more as compared to not moving, p = 0.001); MDRS (OR = 0.794 per transformed unit p = 0.0004). For CA, nine predictors were selected: IgE of house dust mite (OR = 1.308 per log_e _higher, p < 0.0001); IgE of dog (OR = 1.519 per log_e _higher, p < 0.0001); job causing wheezing problems (OR = 13.923 for presence of condition; p < 0.0001); rs8079416 (OR = 0.502 as additive model; p = 0.002); rs11540720 (OR = 0.182 as additive model; p = 0.008); rs5743704 (OR = 0.265 as additive model; p = 0.011); rs11536889 (OR = 0.265 as additive model; p = 0.011); sRaw (OR = 6.509 per log_e _higher; p = 0.0009); MDRS (OR = 0.839 per transformed unit p = 0.013). For DDE, one predictor was selected, the IgE of cat (OR = 1.378 per log_e _higher, p < 0.0001). All features selected by LogitBoost were listed as top-ranked variables by the RF, except for SNPs in the CA outcome. Note that these LR models were obtained from one data set using a single LogitBoost selection, and - given also the degree of correlation among variables - alternative models with equal performance may be selected by varying selection heuristics.

## Discussion

We investigated the ability of linear and non-linear machine learning models to predict asthma, wheezing, and eczema outcomes, according to different operational definitions, with a heterogeneous set of attributes in an adult population. Models were compared in terms of performance, complexity and interpretability. Different feature groups were evaluated and combined in order to understand determinants (and combinations thereof) of asthma symptoms or the presence of eczema. The use of random forests in model building yielded better AUROC, sensitivity and specificity than other methods. This might be due to the ability of random forests to model non-linear functions and account for variable interactions, although the corrected t-test on AUROC did not show a statistically significant difference. Furthermore, the difference between random forests and logistic regression was minimal in predicting asthma phenotypes. In terms of statistical power, however, a larger sample of subjects may reveal undetected differences in the AUROC comparisons of the linear and non-linear methods.

There was a higher prevalence of eczema compared to asthma and wheeze in the population; AUROCs were higher when considering current asthma and current wheeze outcomes (0.84 and 0.76), lower for doctor's diagnosed eczema (0.64). Results show clearly that there is a benefit of merging information from different sources, e.g. lung functions, allergen sensitization tests, genetic markers, demographics and environment. However, in general all models were characterized by a relatively low sensitivity with any feature set combination. A lower sensitivity was obtained compared to that of Chatzimichail *et al*.[23], who used a similar outcome definition: this is because we explicitly excluded any previous personal and familiar diagnosis of asthma, wheeze and eczema from the input set, given the fact that outcomes are often defined recursively on previous episodes. In fact, when utilising previous diagnoses (plus anti-asthma medication usage), sensitivity increased to ≥0.8 (≥0.9 when including anti-asthma medication usage variables) at a minimum specificity of 0.9. However, direct comparison with other methods is only qualitative given the different study designs and populations.

Regarding the importance of features, our findings confirm the important contribution of allergen sensitization (dust mite, dog, cat), along with lung function markers, in predicting asthma diagnoses or symptom patterns. The predictive ability of genetic markers alone is limited, although for the current asthma outcome the LogitBoost algorithm selected a few over the whole set of variables. Our AUROCs for SNPs are in line with the previous estimates of Spycher *et al*.[20], who analysed the genome-wide prediction of childhood asthma and related phenotypes in a longitudinal birth cohort (reporting AUROC of 0.59 for wheeze and of 0.54 for asthma). However, our analysis was not focused on genetic markers: a limited population sample, in terms of the set candidate SNPs as well as of environmental markers, can decrease the power to look for SNP-environment interactions effectively; therefore a more accurate study design is warranted for this objective.

We observed interesting novel and biologically plausible association between bio-impedance and eczema. Previous studies have found that whole body impedance is associated with steroid treatments[45] and several types of cutaneous reactions[46], including an indirect association to Filaggrin-related eczema (via stratum corneum hydration)[47]. Further investigation of this association is warranted.

Limitation of our study include the use of an in-house, rather than externally validated assay for component resolved diagnostics (however, this metric was coupled with validated skin prick testing and blood Immunoglobulin E testing), and the facts that genetic analysis was restricted to candidate genes. Another potential limitation was the naïve policy for missing value imputation; however the extent of missing information was negligible.

## Conclusions

Being a cross-sectional study, with no longitudinal separation of predictors and outcomes, this study is not intended to assess different approaches to causal inference. However, our data demonstrate that even with cross-sectional data, there is considerable scope to build more usefully complex models to better understand asthma and other complex diseases (such as eczema). Future studies might incorporate more factors/attributes and harness longitudinal data in the prediction of later clinical outcomes.

## Competing interests

The authors have no competing interests to declare in relation to this manuscript.

## Authors' contributions

MCFP manuscript writing, machine learning analysis; SM genetic data pre-processing, data collection; AS study design, data collection; AC study design, data management; IEB statistical review. All authors reviewed and contributed to specific sections of the manuscript.

## Declarations

Publication for this article has been funded by grants from J P Moulton Charitable Foundation (sponsoring the MAAS cohort), Medical Research Council (MRC) grants G0601361, MR/K002449/1, University of Manchester's Library via the Research Councils UK (for open-access publications), and by the MRC Health eResearch Centre (HeRC) grant MR/K006665/1.

This article has been published as part of *BMC Medical Genomics *Volume 7 Supplement 1, 2014: Selected articles from the 3rd Translational Bioinformatics Conference (TBC/ISCB-Asia 2013). The full contents of the supplement are available online at http://www.biomedcentral.com/bmcmedgenomics/supplements/7/S1.
